# Replacing Foods with a High-Glycemic Index and High in Saturated Fat by Alternatives with a Low Glycemic Index and Low Saturated Fat Reduces Hepatic Fat, Even in Isocaloric and Macronutrient Matched Conditions

**DOI:** 10.3390/nu15030735

**Published:** 2023-02-01

**Authors:** Jeremy Basset-Sagarminaga, Kay H. M. Roumans, Bas Havekes, Ronald P. Mensink, Harry P. F. Peters, Peter L. Zock, Renée de Mutsert, Jan Borén, Lucas Lindeboom, Patrick Schrauwen, Vera B. Schrauwen-Hinderling

**Affiliations:** 1 Department of Nutrition and Movement Sciences, Maastricht University, P.O. Box 616, 6200 MD Maastricht, The Netherlands; 2 Department of Internal Medicine, Division of Endocrinology and Metabolism, Maastricht University Medical Center, P.O. Box 5800, 6202 AZ Maastricht, The Netherlands; 3 Unilever Food Innovation Center, Plantage 14, 6708 WJ Wageningen, The Netherlands; 4 Department of Clinical Epidemiology, Leiden University Medical Center, P.O. Box 9600, 2300 RC Leiden, The Netherlands; 5 Department of Molecular and Clinical Medicine, University of Gothenburg, P.O. Box 428, 40530 Gothenburg, Sweden; 6 Department of Radiology and Nuclear Medicine, Maastricht University Medical Center, P.O. Box 5800, 6202 AZ Maastricht, The Netherlands; 7 Institute for Clinical Diabetology, German Diabetes Center, Leibniz Institute for Diabetes Research at Heinrich Heine University, 40225 Düsseldorf, Germany

**Keywords:** adults with overweight and obesity, magnetic resonance spectroscopy, heavy water, de novo lipogenesis, dietary intervention, liver glycogen

## Abstract

Background: Current guidelines aim to limit the dietary glycemic index (GI) and intake of saturated fatty acids (SFA). Several studies have shown favorable effects of low-GI or low-SFA diets on intrahepatic lipid content (IHL), but these studies were performed under overfeeding conditions or extreme differences in GI or SFA to maximize the contrast between diets. By combining changes in GI and SFA, we can mimic how people can improve their diet in a realistic setting. Objectives: We investigated the effect on liver fat content and substrate metabolism of both reducing GI and replacing SFA with polyunsaturated fat in practically realistic amounts under isocaloric conditions. Design and Methods: In a randomized crossover study, thirteen overweight participants consumed two diets, one high in GI and SFA (high GI/SFA) and one low in GI and SFA (low GI/SFA) with identical macronutrient composition, for two weeks each. Diets were equal in caloric content, consisted of habitual food items, and had a macronutrient composition that can be easily achieved in daily life. At the end of each intervention, IHL content/composition and liver glycogen were measured by magnetic resonance spectroscopy. Additionally, fasted and postprandial hepatic de novo lipogenesis and glycemic and metabolic responses were investigated. Results: IHL was significantly lower (−28%) after the two-week low-GI/SFA diet (2.4 ± 0.5% 95% CI [1.4, 3.4]) than after the two-week high-GI/SFA diet (3.3 ± 0.6% 95% CI [1.9, 4.7], *p* < 0.05). Although hepatic glycogen content, hepatic de novo lipogenesis, hepatic lipid composition, and substrate oxidation during the night were similar between the two diets, the glycemic response to the low-GI/SFA diet was reduced (*p* < 0.05). Conclusions: Changes in macronutrient quality can already have drastic effects on liver fat content and postprandial glycemia after two weeks and even when energy content and the percentage of total fat and carbohydrate remains unchanged.

## 1. Introduction

Obesity is a major health problem worldwide and its prevalence continues to rise [1]. In fact, in the last 45 years, obesity rates nearly tripled [1]. Obesity has been associated with excessive fat storage in the liver, referred to as Non-Alcoholic Fatty Liver (NAFL). In obese people, NAFL prevalence rates as high as 50–70% have been reported [2]. NAFL increases the risk of developing type 2 diabetes mellitus two- to three-fold, even when corrected for BMI [3,4]. A healthy diet is a cornerstone in the prevention and treatment of obesity and related metabolic conditions such as NAFL. Low-carbohydrate and low-fat diets have both been shown to be effective in reducing liver fat in overweight individuals [5,6,7,8].

Importantly, not only does the percentage of energy (En%) from dietary fat and carbohydrates seem to be of significant importance but also their quality [9,10,11,12,13]. Specifically, the saturation of the fatty acids and the glycemic index (GI), indicating to what degree the carbohydrates in food affect blood glucose levels, can influence liver fat content. Diets high in saturated fatty acids (SFA) have been shown to increase liver fat content when compared to diets high in poly-unsaturated fatty acids (PUFA) [10,11,12,14]. Furthermore, a high-GI diet has been shown to increase liver fat content as compared to a low-GI diet [13]. These studies were, however, performed under overfeeding conditions and/or used more extreme differences in macronutrients (e.g., 71 En% carbohydrate and 14 En% total fat [13]) and were therefore not comparable to dietary patterns of the general population and did not reflect realistic food choices [15]. Thus, it remains unclear whether changes in food choices that can be performed by individuals to improve their diet in terms of lowering the GI and lowering SFA intake compared to a high-GI/SFA diet are beneficial for liver fat content. We investigated this in the setting of dietary content reflecting typical dietary patterns of the general population. Combining low GI/low SFA on the one hand and high GI/high SFA on the other hand, however, gives us means to increase the contrast between the two diets while keeping the diets within the realistic setting of how individuals can improve their diet, even without changing macronutrient composition. Whether such a ‘healthy diet’ low in GI and SFA indeed reduces liver fat content as compared to an ‘unhealthy’ diet high in GI and SFA has not been addressed yet.

In addition, the underlying pathways involved in the modulation of liver fat content by such diets are of interest. Unraveling these pathways could help identify targets that can be used to treat NAFL. For example, it has been suggested that the type of fatty acid may affect whole-body fat oxidation. Stable isotope studies show that exogenous SFA is oxidized to a lesser extent than unsaturated fatty acids [16,17,18] and acute meal challenge studies have reported lower whole-body fat oxidation with SFA compared to monounsaturated fat (MUFA) [19,20], increasing the risk of ectopic fat accumulation in normally lean organs such as the liver. High-GI diets, on the other hand, could induce liver fat accumulation through increased glycemic and insulinemic response [13]. High insulin and high glucose levels can lead to increased hepatic glycogen stores [13] and promote de novo lipogenesis and reduce fat oxidation. Therefore, simultaneously lowering the SFA content and GI of the diet may be a strong candidate for dietary manipulation of hepatic fat content.

Here, we investigated whether consumption of a low-GI/SFA diet for two weeks, compared with an isocaloric high-GI/SFA diet, reduces liver fat content in overweight individuals. In addition, we studied whether decreases in liver fat content were parallel to an increase in whole-body fat oxidation, a decreased glycemic response, reduced hepatic glycogen levels, reduced hepatic de novo lipogenesis, and a decreased saturated hepatic fat fraction.

## 2. Materials and Methods

This study was conducted at Maastricht University Medical Center, the Netherlands, between August 2019 and March 2021, and was approved by the Medical Ethical Committee of Maastricht University Medical Centre. The research was performed in accordance with all relevant ethical regulations regarding human research participants. The clinical trial registration number is NCT04054297 (ClinicalTrials.gov https://clinicaltrials.gov/ct2/show/NCT04054297 (accessed on 20 January 2023)).

### 2.1. Participants

All participants recruited for this study provided written informed consent. Thirteen overweight/obese (BMI 27–38 kg/m^2^) volunteers, aged 45–75 years, were recruited for this study. Participant characteristics are shown in Table 1. Female study participants were postmenopausal. Exclusion criteria were engagement in vigorous exercise for more than 2 h per week, unstable body weight (weight loss or gain more than 3 kg in 3 months preceding enrollment), alcohol consumption of more than 2 units per day, smoking, contra-indication for MRI, being vegetarian, vegan or food intolerance to common foods, use of medication known to interfere with the outcome parameters, and diabetes or other active diseases.

### 2.2. Research Design and Methods

**Figure 1:**
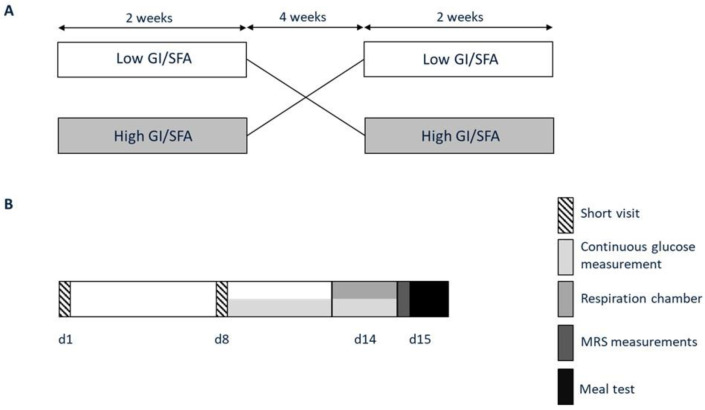
Study protocol. (**A**) Participants participated in two dietary interventions in a randomized order; a two-week high-GI/SFA diet and a two-week low-GI/SFA, interspersed by a four-week washout. (**B**) At the end of each dietary intervention, volunteers visited the university for study measurements (days 14–15). At the start and after one week of each of the intervention periods, participants visited the research facilities for instructions and body weight and food intake evaluation (short visits d1 and d8).

**Figure A1:**
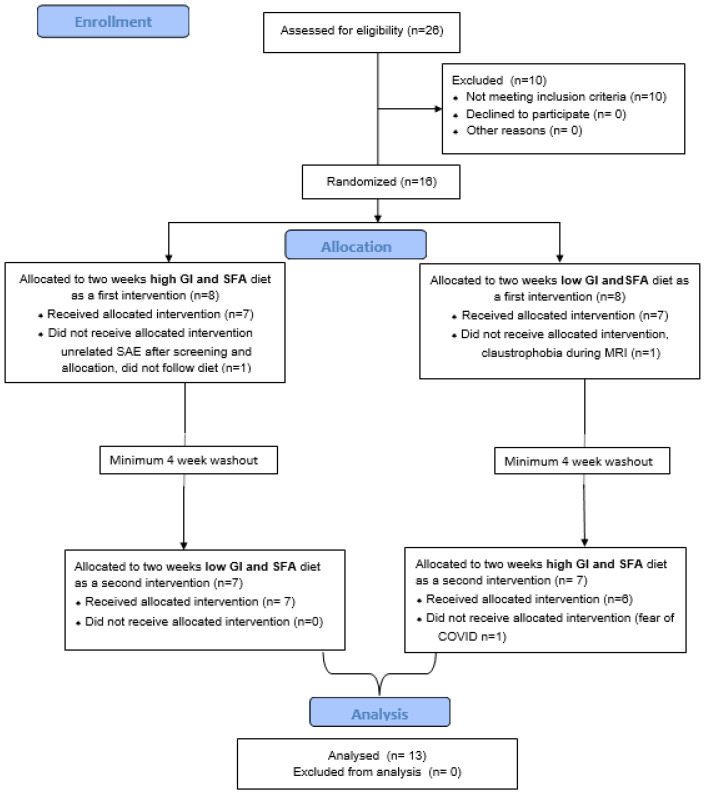
Participant recruitment flowchart.

Volunteers participated in a randomized crossover trial (participant flowchart Appendix A Figure A1), consisting of a two-week high-GI/SFA diet and a two-week low-GI/SFA diet (Figure 1) separated by a washout period of at least 4 weeks with testing at the end of each period. After screening for eligibility, participants were randomly assigned to start with either a low- or high-GI/SFA diet by use of an online randomizer. Dietary interventions started with a short visit to the research facilities for body weight assessment. During the entire study period, no changes in lifestyle were allowed, except for the change in dietary composition as part of the study design. Participant characteristics such as weight and BMI were checked after the washout period to ensure that they did not differ from the baseline. 

### 2.3. Diets

At the start of the dietary periods, individual energy requirements were estimated using Harris–Benedict equations adjusted for the physical activity level (PAL), which was low by design, and age [21]. For <50 year, 50–70 year, and >70 year, a PAL value of 1.5, 1.4, and 1.3 was used, respectively. Dietary menus were designed for each intervention by a dietician in collaboration with the researchers. Diets were comparable in total macronutrient content (En% derived from carbohydrates, fats, protein in low GI/SFA: 53 ± 0.7%, 27 ± 0.8%, 15 ± 0.6%; high GI/SFA: 55 ± 1.7%, 30 ± 0.7%, 15 ± 0.3%), but differed in fatty acid composition and GI. SFA represented 5% and 15% of the total energy content of the diet for low- and high-GI/SFA diets, respectively. The average GI of the diets was 36 and 60 for the low- and high-GI/SFA diets, respectively. The GI tables of Foster–Powell were used to estimate GI [22]. 

Participants were provided with a clear daily menu/checklist indicating ingredients (with pictures), their quantity in grams (they were provided a food scale if needed), and instructions on how to cook them. These menus also contained a checklist to check for adherence to the diets. Furthermore, participants were urged to adhere to the dietary menus in order to maintain body weight throughout the study and keep track of their food intake through dietary checklists. All products with a long shelf-life were provided at the start of each diet (e.g., canned foods, pasta, rice, packaged soups). Participants were instructed to buy other prescribed food products, such as fresh fruit and vegetables, as indicated by an individual food-product list, and participants checked off the items when consumed. Dietary content and composition and their adherence to the intervention were monitored by evaluations of the dietary checklist and body weight after one week, when volunteers returned for new food supplies and the weight check and at the end of the dietary interventions. Additionally, continuous glucose monitoring (CGM) could have also aided the adherence to the diets by being “monitored” during the entire second week of each diet. In the case of weight loss or gain after one week, the researcher provided additional counselling and, if necessary, the number of calories provided was adapted to ensure energy balance. 

### 2.4. Measurement of Hepatic Fat Content and Composition

On day 15, at 7:15 a.m. after an overnight fast, the hepatic lipid content and lipid composition (the fraction of hepatic SFA, MUFA, and PUFA) were determined by 1H-MRS on a 3T MR system (Achieva 3T-X Philips Healthcare, Best, The Netherlands) using a STEAM sequence (repetition time (TR) of 4500 ms/echo time (TE) of 20 ms/mixing time (TM) of 16 ms, spectral bandwidth of 2000 Hz, and 2048 data points) and fat content and fat composition was determined as described earlier [23]. Lipid content was calculated after T2 correction as the ratio of the CH2 peak relative to the sum of the unsuppressed water resonance and CH2 peak and converted to weight/weight percentage. 

### 2.5. Measurement of Hepatic Glycogen

On day 15 at 6:30 a.m. after an overnight fast, hepatic glycogen was determined by 13C-MRS. All 13C-MRS experiments were performed on a 3T MR system (Achieva 3T-X Philips Healthcare, Best, The Netherlands) using a 21 × 24 cm 13C quadrature detection surface coil (RAPID Biomedical GmbH, Rimpar, Germany). Hepatic glycogen levels were measured as described previously [24] by acquiring 13C MR spectra (Free induction decay, using block pulses calibrated to reach a 90-degree pulse at an 8 cm depth; TR: 280 ms; NSA: 4096). Data analysis was performed with an in-house-developed Matlab script including automatic postprocessing and integration of spectra and corrections for the coil sensitivity profile, as reported before [24]. Liver volume was measured directly after the hepatic glycogen measurements by MRI. Analyses were performed manually on cross-sectional MRI images in MATLAB. Relative changes in glycogen arbitrary units (AUs) between the high-GI/SFA and low-GI/SFA measurement ((hepatic glycogen low GI/SFA − hepatic glycogen high GI/SFA)/hepatic glycogen high GI/SFA × 100) were calculated and are reported in the results section as a single value for the relative change between high and low GI/SFA. Total hepatic glycogen was calculated as the hepatic glycogen × liver volume.

### 2.6. Respiration Chamber Measurement

Participants stayed in a respiration chamber on day 14 from 7:30 a.m. onwards. The metabolic chamber is a small room with basic amenities in which oxygen consumption and carbon dioxide production were measured continuously in sampled room air by whole-room indirect calorimetry (Omnical, Maastricht Instruments, Maastricht, The Netherlands) [25]. Volunteers received all meals matching their assigned diet. During the overnight stay in the respiration chamber, the sleeping metabolic rate, respiratory exchange ratio, fat oxidation, and carbohydrate oxidation were assessed. Participants were instructed to go to sleep at 11:00 p.m. At 5:45 a.m. the next morning, participants were woken up and left the chamber in an overnight fasted state.

### 2.7. Meal Test

After the MRS measurements on the morning of day 15, a meal test was performed. To this end, a breakfast (20% of daily energy needed) matching the assigned diet was provided to the participants at 8:30 AM (t = 0). Before the meal (t = −15) and at t = 45, t = 75, t = 135, t = 195, and t = 255 min, indirect calorimetry measurements using a ventilated hood system (Omnical, Maastricht Instruments, Maastricht University) were performed. VO2 and VCO2 were used at these timepoints to assess the energy expenditure, respiratory exchange ratio (RER), and fat and carbohydrate oxidation. 

### 2.8. Plasma Analysis

Blood collected in EDTA-coated tubes was immediately stored on ice, centrifuged, and plasma was stored at −80 °C until analyses Blood collected in serum tubes was stored at room temperature for at least 30 min to allow coagulation, followed by centrifugation and storage at −80 °C until analyses. Glucose (Horiba, Montpellier, France), FFA (WAKO, Neuss, Germany), and triglycerides (Sigma, St. Louis, MI, USA) levels were measured colorimetrically using a Cobas Pentra C400 analyzer (Horiba, Montpellier, France). All samples from one participant were analyzed within the same run.

### 2.9. Continuous Glucose Monitoring

During the short visit after 1 week of the diet, a continuous glucose monitoring sensor (FreeStyle Libre, Abbott GmbH & Co, Wiesbaden, Germany)was placed at the back of the upper arm. Interstitial fluid glucose was measured every 15 min by the sensor. Measurements took place up to day 15 when the sensor was removed in the morning before the MRS measurements. Glycemic variability was calculated as the average coefficient of variation (%CV).

### 2.10. Body Composition

Body mass and body volume were assessed using air-displacement plethysmography (ADP) using the Bod Pod device (Cosmed, Italy, Rome) according to the manufacturer’s instructions [26]. Thoracic gas volume was predicted based on equations included in the Bod Pod software (version 4.2.0). From these data, the body fat percentage was calculated as described by Siri [27].

### 2.11. Deuterated Water Measurement of Hepatic DNL

A background blood sample was drawn in the afternoon before intake of the deuterated water. Together with the evening meal, participants were given 2.86 g/kg body weight of deuterated water (70% 2H_2_O, Cambridge Isotope laboratories) in two servings. A blood sample for DNL analysis was drawn at fasting in the morning, 16 h after the first serving of deuterated water and in the postprandial state, and 4 h after the breakfast meal. The DNL was analyzed via enrichment in VLDL-TG of deuterated water [28,29].

### 2.12. Calculations

Energy expenditure was calculated based on the measured averaged oxygen and carbon dioxide concentrations in the inspired and expired gases with the assumption that protein oxidation was negligible, using the Weir equation [30]. The sleeping metabolic rate was calculated as the lowest average 3 h energy expenditure during the sleeping period. Glucose oxidation and fat oxidation rates were calculated according to the Brouwer equation [31].

### 2.13. Statistical Analyses

The primary outcome parameter was the difference in IHL between the two diets. Exploratory outcome parameters included the effect of the two diets on DNL, 24-h glycemic response, hepatic glycogen content, plasma BHB levels, hepatic lipid composition, plasma metabolites related to energy expenditure, energy expenditure, and substrate oxidation.

The sample size of *n* = 13 was calculated to detect potential differences in the primary outcome: Differences in IHL between the two diets. Based on a similar study making use of changes in GI [13], the absolute difference in liver fat content change (an absolute increase of 1.3 percentage points after high GI and a decrease of 0.4 percentage points after low GI) between the two diets is 1.7 percentage points, with an SDdif of 0.8 percentage points, resulting in an effect size of 2.13%. This effect size is comparable or higher regarding other studies investigating the effects of dietary fatty acid composition on liver fat content (Bjermo 2012 et al.: abs. dif 1.2%, SDdif 0.4%, effect size 3.0, Rosqvist 2014 et al.: abs. dif 0.52%, SDdif 0.23%, effect size 2.3, Luukkonen et al. 2018: abs. dif 2.0%, SDdif 0.3%, effect size 6.7) [10,11,12]. As these dietary intervention studies used differences in macronutrient content and/or overfeeding, the expected difference was assumed to be smaller in the current study. Therefore, we assumed an effect of 40% of the effect found by the study by Bawden et al. [13] with a difference of 0.68 percentage points (absolute liver fat content). The effect size was then accordingly determined to be 0.85, and using an α of 0.05 and 1-β of 0.8, 13 subjects needed to be included in a crossover design for sufficient statistical power.

Results were expressed as mean ± SEM. Participant characteristics were expressed as mean ± SD. Continuous variables were tested for normality (Shapiro–Wilk test). In case variables were not normally distributed, non-parametric statistical tests were used. Changes in liver fat content/composition and glycogen between diets were assessed by a paired-sample *t*-test. 

Changes across the time course during the meal test after both diets and changes across the time course during the continuous glucose measurements on day 14 were assessed using a two-way repeated-measures ANOVA to evaluate any significant effect of diet and timepoint and any interaction between diet and timepoint. A *p*-value < 0.05 was considered statistically significant. Statistical analyses were performed using SPSS 27.0 for Windows.

## 3. Results

### 3.1. Liver Fat Content, Liver Fat Fraction, Body Weight, and Adherence

**Figure 2:**
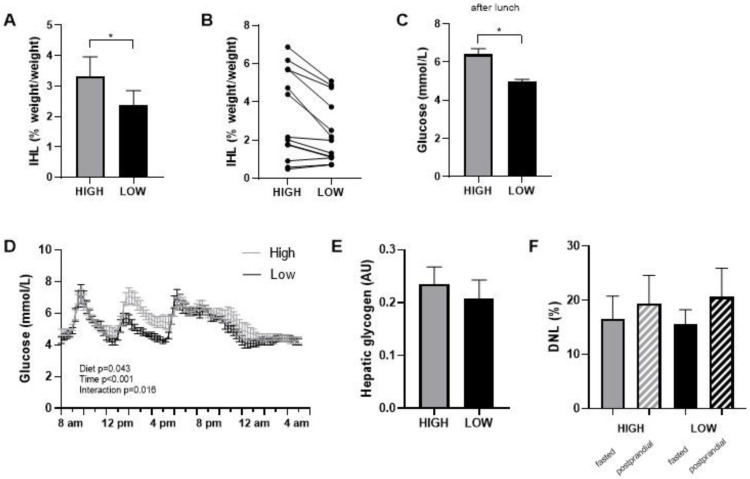
Intrahepatic lipid content (IHL) after a two-week high- or low-GI/SFA diet. (**A**) IHL content mean ± SEM. (**B**) individual IHL data (*n* = 13). (**C**) Average glucose in interstitial fluid over the 3 h following lunch (*n* = 10). (**D**) Continuous glucose measurements during standardized day 14 in the respiration chamber, after two weeks of a low-GI/SFA or high-GI/SFA diet. (**E**) Hepatic glycogen comparison after the low and high-GI/SFA diets, arbitrary units (AU) (*n* = 10). (**F**) DNL as the percent of newly synthesized palmitate within the VLDL fraction in fasted state and after a 4 h meal test (postprandial). * *p* < 0.05.

Food checklists completed during the intervention periods indicated that all participants closely adhered to the prescribed diets. This was also supported by consulting with the participants during the weekly checkups. Body weight slightly decreased in both diets during the first week and stabilized thereafter (in the high-GI/SFA diet, pre; 88.4 ± 3.9 kg, post; 87.5 ± 3.8, *p* < 0.05 and in the low-GI/SFA diet, pre; 88.3 ± 3.9 kg, post; 87.2 ± 3.8, *p* < 0.05). The change in body weight was, however, similar in both diets (High GI/SFA; −0.8 ± 0.4 kg, Low GI/SFA; −1.2 ± 0.3 kg, *p* > 0.05), and body weight at the start of each diet was not significantly different (*p* > 0.05). Intrahepatic lipid content (IHL) was significantly lower (−28%) after the two-week low-GI/SFA diet (2.4 ± 0.5%, 95% CI [1.4, 3.4]) than after the two-week high-GI/SFA diet (3.3 ± 0.6%, 95% CI [1.9, 4.7], *p* < 0.05, Figure 2A). Four subjects had an IHL content of >5% following the high-GI/SFA diet (qualifying for NAFL), and in these four subjects, the IHL content was below 5% following the low-GI/SFA diet (Figure 2B). The change in body weight was not correlated to the change in IHL content (*p* > 0.05). In terms of fatty acid composition, no significant differences were found in the fractions of SFA (*n* = 7, High GI/SFA: 43.1 ± 1.4%, Low GI/SFA: 41.6 ± 3%, *p* > 0.05), MUFA (*n* = 6, High GI/SFA: 36.9 ± 1.6%, Low GI/SFA: 40.3 ± 2.6%, *p* > 0.05), or PUFA (*n* = 6, High GI/SFA: 21.1 ± 1.4%, Low GI/SFA: 20.4 ± 2.2%, *p* > 0.05). Due to some subjects having low liver fat (<2%) or breathing artifacts during the scan, the quality of the signal was not sufficient to calculate the liver fat fraction in *n* = 7 individuals for MUFA and PUFA fractions and *n* = 6 for the SFA fraction. 

### 3.2. Glycemic Response, Fasted Hepatic Glycogen, and Hepatic De Novo Lipogenesis 

Interstitial glucose levels were measured with a continuous glucose monitor (CGM) during the second week of each diet. Glycemic variability, calculated as the average %CV of glucose over the whole week, was significantly higher in the high GI/SFA diet compared to a low-GI/SFA diet (*n* = 10, High GI/SFA 20.4 ± 1.4%; Low GI/SFA 17.8 ± 1.4%, *p* < 0.05). On day 14, the last day of the dietary period, participants followed a standardized test day in our facilities. A two-way repeated measures ANOVA revealed a significant diet ×time interaction (*p* < 0.05, Figure 2D) on interstitial glucose levels on day 14. Differences in the glycemic response were especially apparent after lunch, with significantly lower glucose levels in the low-GI/SFA diet over the 3h following lunch (*p* < 0.05, Figure 2C). 

There was no significant difference in hepatic glycogen content after the high compared to the low GI/SFA diet (−5.2 ± 11.6%, *p* > 0.05, Figure 2E). Furthermore, no changes were observed in total hepatic glycogen corrected for liver volume (hepatic glycogen × * liver volume). Liver volume, however, was slightly higher in the high-GI/SFA diet (1375 ± 81 cm^3^) when compared to the low-GI/SFA diet (1312 ± 79 cm^3^, *p* < 0.05). Changes in IHL did not significantly correlate with changes in liver volume (Pearson r = 0.541, *p* > 0.05). As expected, de novo Lipogenesis was significantly higher in the postprandial state than the fasted state for both diets (*p* < 0.05); however no changes in fasting or postprandial DNL were observed between the two diets (Figure 2F).

### 3.3. Substrate Oxidation

**Figure 3:**
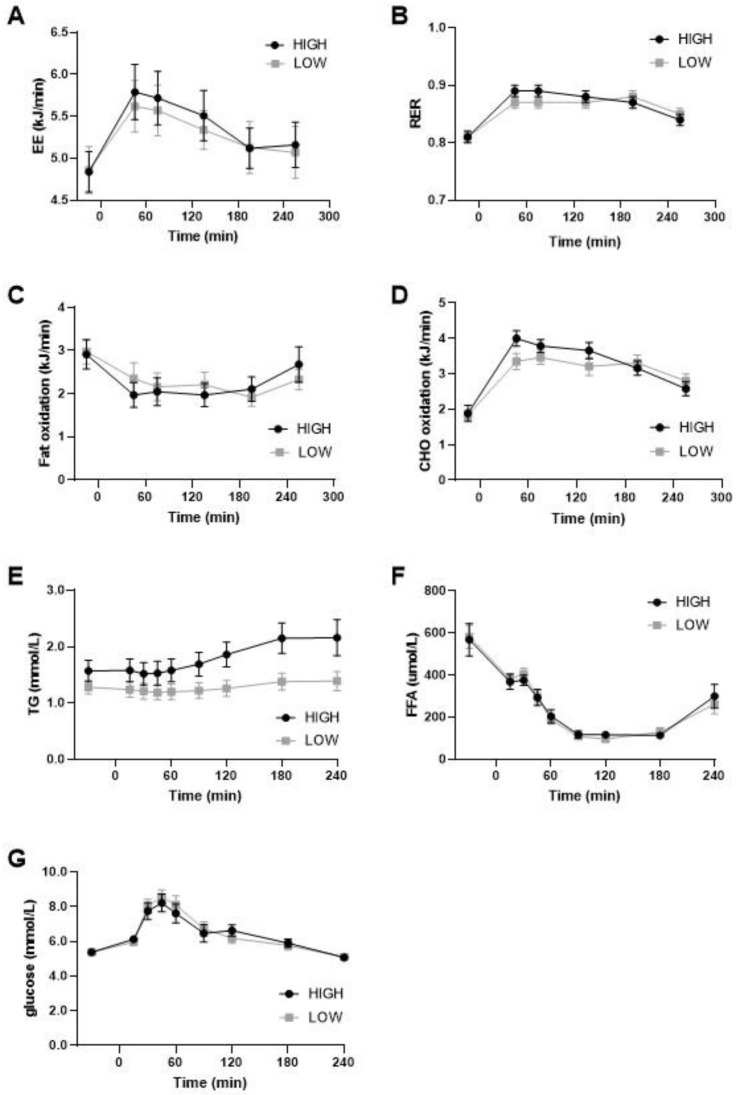
Energy expenditure, substrate oxidation, and plasma values during meal test after two weeks of a high- or low-GI/SFA diet. (**A**) Energy expenditure (EE). (**B**) Respiratory exchange ratio (RER). (**C**) Fat oxidation. (**D**) Carbohydrate (CHO) oxidation during the meal test after two weeks of a high- or low-GI/SFA diet (*n* = 10). (**E**) Plasma triglycerides levels, significant effect of diet and diet × time *p* < 0.05. (**F**) Plasma-free fatty acids. (**G**) Plasma glucose (*n* = 10).

On day 14, substrate oxidation was postprandially measured during daytime and nighttime in a respiration chamber and the following morning (day 15) using a ventilated hood after a breakfast meal. The sleeping metabolic rate (SMR) during the night was not different between the diets (Low GI/SFA: 4.37 ± 0.19 Kj/min; High GI/SFA: 4.36 ± 0.17 Kj/min; *p* > 0.05), nor were carbohydrate oxidation during SMR (low GI/SFA: 2.32 ± 0.15 Kj/min; High GI/SFA: 2.38 ± 0.19; *p* > 0.05) or fat oxidation during SMR (Low GI/SFA: 2.12 ± 0.14 kJ/min; High GI/SFA: 2.06 ± 0.25 kJ/min; *p* > 0.05). On day 14, in the respiration chamber, no significant differences in the respiratory exchange ratio between the diets were found during daytime (11:00 to 22:00) (Low GI/SFA: 0.85 ± 0.01; High GI/SFA 0.86 ± 0.01; *p* > 0.05) or during nighttime (00:00 to 5:00) (Low GI/SFA: 0.84 ± 0.01; High GI/SFA 0.85 ± 0.01; *p* > 0.05). Moreover, under fasting conditions in the morning, substrate oxidation was similar (Figure 3B) as measured by the ventilated hood. Postprandially, after a breakfast matching the corresponding diet, the peak in carbohydrate oxidation was lower after the low GI/SFA diet, but this was not statistically significant (Figure 3D). Fat oxidation seemed to increase in the first two hours after the test meal in the low-GI/SFA diet, but this was also not significant (*p* > 0.05, Figure 3C). Total energy expenditure was also not significantly different between the two diets under fasting conditions (*p* > 0.05, Figure 3A) or after the corresponding breakfast meal (*p* > 0.05, Figure 3A).

### 3.4. Plasma Triglycerides, Free Fatty Acids, and Glucose during Meal Test

We measured the changes in plasma triglycerides (TG), free fatty acids (FFA), and glucose in response to the meal test. A significant effect of diet and the diet × time interaction was found for plasma TG (*p* < 0.05; Figure 3E). TG was significantly higher in the high-GI/SFA diet when compared to the low-GI/SFA diet in the fasted state (*p* < 0.05), and also at all other timepoints (*p* < 0.05, Figure 3E). No significant effect of diet × time was found in plasma glucose and FFA (Figure 3F,G). 

## 4. Discussion

It has previously been shown that macronutrient quality (i.e., GI of carbohydrates and saturation of fats) can influence liver fat storage [9,10,11,12,13,14,32]. These studies were, however, performed under overfeeding conditions and/or used extreme differences in GI or fat saturation. Whether dietary GI and SFA content is of importance in modulating liver fat content when consuming diets fitting the dietary patterns of the general population was yet unknown. Here, we showed that a two-week isocaloric low-GI/SFA diet compared to a high-GI/SFA diet, with similar total fat and carbohydrate content, reduces liver fat content and, as expected, lowers the glycemic response. Hepatic glycogen, hepatic de novo lipogenesis, and whole-body substrate oxidation were not significantly different between diets.

We show that IHL was 28% lower after a two-week low- compared to a high-GI/SFA diet. This is in line with previous studies investigating the effects of either differences in dietary GI or differences in dietary fat saturation [10,11,12,13,14,32]. To make sure that our results are relevant to daily life, we used normal food items (Table A1, Appendix A) and a macronutrient composition that is typically consumed by the general population. The results indicate that carbohydrate and fat quality have important effects on IHL storage when consumed within normal ranges of dietary intake. Interestingly, four out of the thirteen participants had an IHL content of more than 5% following the high-GI/SFA diet (Figure 2B), which would classify them as having NAFL according to the diagnosis criteria [33]. However, in all four of these participants, the low-GI/SFA diet led to IHL content lower than 5%, and they would therefore not be classified as having NAFL. This was achieved in only two weeks in isocaloric conditions, without changing the total amount of carbohydrates and fat, which highlights the importance of the quality of macronutrients in the development of fatty liver. 

We hypothesized that a lower glycemic response could underlie improvements in IHL. 

Indeed, we found a significant effect of diet (*p* < 0.05) and the diet × time interaction (*p* < 0.001) on glycemia on day 14 (Figure 2D), the standardized test day. This was especially apparent after lunch (*p* < 0.05; Figure 2C). This is in line with findings from Bergia et al. [34] who reported seeing more robust differences in glycemia between a high- vs. low-GI meal test after the lunch meal when compared to the breakfast. 

Glycemic variability, measured as the coefficient of variation (%CV), over four weekdays was significantly lower during the low-GI/SFA diet when compared to the high-GI/SFA diet. This finding is in line with previous studies showing that a higher-glycemic-index diet significantly increased glycemic variability [34,35]. Monitoring glycemic variability was also a good way to check participants’ adherence to the diet, and our findings confirm that participants indeed adhered to the high-GI or low-GI diets. Previously, differences in the glycemic response have been observed acutely in healthy volunteers after consuming low- compared to high-GI meals [13,34,36]. However, the GI of the high-GI diets was higher in these studies.

One of the principal pathways leading to increases in IHL is hepatic de novo lipogenesis [37]. With increases in glycemia, we expected DNL to be higher in the high-GI/SFA diet when compared to the low-GI/SFA diet. Additionally, dietary PUFAs such as omega-3 fatty acids have been shown to decrease hepatic DNL [38], and one could therefore have expected a reduced DNL in the low-GI/SFA diet. We however did not find any differences in DNL between the two diets, neither in the fasted nor the postprandial state. Our findings are in line with a recent study by Parry et al. [32], where they did not find a significant change in fasted DNL even after 4 weeks of a diet enriched in either SFA or sugar. They also did not find any changes in postprandial DNL [32]. It is possible that the intervention period was not sufficient to induce changes in fasting DNL. Regarding postprandial DNL, most studies showing differences in postprandial DNL have compared hypercaloric diets or high-carbohydrate diets. It is therefore possible that, in our study, the differences in GI were not sufficient to elicit higher rates of postprandial DNL since total fat, carbohydrates, and energy content were similar in both diets [11,39,40,41]. Under the conditions of energy balance, it could be that other pathways such as glycogen synthesis and glucose oxidation are sufficient to dispose of carbohydrates [32]. In line with our findings regarding DNL, no statistically significant changes in the liver fat fractions of SFA, MUFA, or PUFA were found. The hepatic saturated fat fraction has previously been shown to be associated with DNL [23] since DNL principally leads to the synthesis of SFA and not PUFA [23]. 

Another potential mechanism explaining the reduced IHL and lower glycemic response may involve changes in hepatic glycogen levels. Filled hepatic glycogen stores reduce the need for glucose entering the liver in the postprandial state to be used for glycogen storage, and as a result, glucose can be directed towards DNL [42]. Furthermore, reduced hepatic glycogen stores force substrate oxidation towards higher fat oxidation levels. Therefore, strong fluctuation in hepatic glycogen is believed to be key for good metabolic health. In contrast to our expectations, we did not observe changes in fasting liver glycogen content between diets. It is possible that any effect of the diet on glycogen stores was masked by the effect of the overnight fast on liver glycogen. Nevertheless, liver volume was slightly but significantly reduced after the low- compared to the high-GI/SFA diet. It is possible that these changes in liver volume were due to small changes in glycogen that could not be detected with the 13C-MRS glycogen measurement. Alternatively, the decrease in IHL content could also have played a role in the reductions in total liver volume after the low-GI/SFA diet. 

Similar to hepatic glycogen, no differences were found in whole-body substrate oxidation during the night after two weeks of a low- compared to a high-GI/SFA diet. As there were no pronounced differences in hepatic glycogen content on a low- compared to a high-GI/SFA diet, there was possibly no trigger to increase overnight fat oxidation in the low-GI/SFA diet. After a breakfast test meal in the low-GI/SFA diet, substrate oxidation seemed to shift from carbohydrate to fat oxidation in the first two hours postprandially. However, the differences did not reach statistical significance. As expected, total energy expenditure was also not significantly different after the two corresponding breakfast meals (Figure 3A) since energy content and macronutrient percentages were matched. In addition to GI, the saturation of dietary fat could also have been important in determining postprandial substrate oxidation. Meal challenge experiments have shown increased whole-body fat oxidation with unsaturated fatty acids compared to saturated fatty acids when investigated acutely [19,20] or after a one-week diet [43]. Therefore, the increased SFA content in the high-GI diet would be expected to increase postprandial carbohydrate oxidation further. Some of these studies investigated the effects of dietary fat saturation in diets containing 40 En% of fat (vs. 27–30 En% fat in this study), thereby maximizing the potential to modulate whole-body fat oxidation. This might explain the more pronounced effects in these studies as compared to our results. It should also be noted that we investigated fat oxidation at the whole-body level. To investigate if increased fat oxidation contributed to decreased IHL, we would need to determine if liver-specific fat oxidation is changed by dietary GI/SFA content. 

Next to the possible pathways that could underlie the changes in IHL investigated here (DNL and fat oxidation), other pathways may also contribute to liver fat accumulation. For example, FFA uptake, dietary fat storage, and VLDL-TG secretion could be influenced by dietary GI/SFA content and should be considered in future studies. Luukkonen et al. showed that overfeeding saturated fatty acids increased adipose tissue lipolysis while overfeeding unsaturated fatty acids decreased it [11]. Because adipose tissue lipolysis is the main determinant of hepatic FFA uptake and approximately 60% of liver lipids are derived from FFA uptake [37,44], it is conceivable that the effects on IHL found here are at least partly mediated by changes in FFA uptake as a result of differences in the saturation of the dietary fatty acids. We did not directly investigate adipose tissue lipolysis here; however, FFA levels in the fasting state or postprandially were not different between the two diets. 

To our knowledge, this is the first study examining the combined effect of reducing both GI and SFA content on IHL in a diet that can be achieved in real-life with habitual foods. Remarkably, IHL was already reduced after two weeks’ time. Moreover, the changes in IHL were not related to weight differences. The intervention diets did not differ in energy content and total fat and carbohydrates, which highlights the importance of macronutrient quality in the diet. Such dietary changes can be much more feasible for people at risk than implementing rigorous dietary plans aimed at weight loss. This reduction in IHL was accompanied by a lower glycemic response as measured by CGM during the standardized day 14 in the respiration chamber (Figure 2D), which might underlie the change in liver fat content. 

### Limitations

Despite the dietary counselling and adaptations made to dietary energy content in case volunteers lost weight in the first week, body weight was reduced by, on average, ~0.8–1.2 kg in both diets. However, body weight was reduced to a similar extent in both diets. The small difference of 0.4 kg between the interventions is expected to lead to a very minor decrease in liver fat of <0.3%, which is much smaller than the differences found in the present study [45]. Furthermore, the difference in IHL content observed after the two diets did not correlate with the differences in body weight. 

In the present study, a clear distinction in the glucose pattern could especially be made upon lunch and also after dinner as measured by CGM. The specific menus used on day 14 of the interventions clearly differed in GI for lunch (33 vs. 57), dinner (43 vs. 63), and after-dinner snack (33 vs. 64). The GI of the breakfast meals provided to the volunteers on day 14 were, however, less distinctive (45 vs. 52), which could explain why the expected differences in glycemic response are not seen after breakfast. 

We recognize that the sample size in this study was limited, which could potentially explain the lack of findings with respect to exploratory outcomes such as de novo lipogenesis and liver glycogen content. The analysis of specific liver fat composition requires high-quality spectra to allow for the calculation of percentage PUFA and MUFA. In the current study, several measurements of IHL composition had to be excluded due to a low signal-to-noise ratio, especially in volunteers with very low liver fat content. In populations with higher liver fat content, a bigger signal is available, thus leading to better quality. 

## 5. Conclusions

Changes in macronutrient quality can have drastic effects on liver fat content and postprandial glycemia after just two weeks and even when energy content and percentage of total fat and carbohydrate remain unchanged, showing the power of making more healthy choices to minimize GI and limit SFA intake. The exact mechanisms underlying these reductions in IHL remain to be elucidated. Other pathways contributing to liver fat accumulation, (i.e., FFA uptake, dietary fat storage, and VLDL-TG secretion) could be influenced by dietary GI/SFA content and should be considered in future studies.

## Figures and Tables

**Table 1:** Subject characteristics.

	Participants (*n* = 13)
Age (years)	67 ± 6
BMI (kg/m^2^)	29.5 ± 1.9
Sex (f/m)	5/8
Total body fat (%) f/m	44.4 ± 6.1/32.3 ± 3.6
Plasma glucose (mmol/L)	5.4 ± 0.4
Plasma TG (mmol/L)	1.4 ± 0.6
ALT (U/L)	27 ± 11
AST (U/L)	26 ± 8
Gamma-GT (U/L)	31 ± 23

Data are presented as mean ± SD, *n* = 13.

## Data Availability

Data will be made available upon reasonable request.

## Appendix A

**Table A1:** Example diets.

High GI/SFA	Low GI/SFA
Breakfast	
Tiger bread Nutella Full fat milk Orange juice	Oats Semi skimmed milk Banana Dutch breakfast bread
Lunch	
Tiger bread Ham Cheese slices Banana Tomato soup with meatballs (packaged)	Tomatoes Hummus Rye bread Cucumber Apple
Dinner	
Mashpot mashed potatoes with vegetable and smoked sausage Creamy cottage cheese	Pasta salad with chicken Cottage cheese
